# A single-center analysis of visual outcomes and associated factors after intravenous methylprednisolone treatment for dysthyroid optic neuropathy

**DOI:** 10.1186/s12886-023-02789-5

**Published:** 2023-01-23

**Authors:** Parinee Kemchoknatee, Duanghathai Tangon, Thansit Srisombut

**Affiliations:** 1 Department of Ophthalmology, Rangsit University, Rajavithi Hospital, 2 Phaya Thai Rd, Thung Phaya Thai, Ratchathewi, Bangkok, 10400 Thailand; 2 Faculty of Medicine Rajavithi Hospital, Rangsit University, 2 Phaya Thai Rd, Thung Phaya Thai, Ratchathewi, Bangkok, 10400 Thailand

**Keywords:** Dysthyroid optic neuropathy, Graves orbitopathy, Thyroid eye disease, Intravenous glucocorticoids. Intravenous methylprednisolone

## Abstract

**Background:**

Dysthyroid optic neuropathy (DON) is a serious threatening vision loss in Graves’ ophthalmopathy (GO). Although the European Group on Graves’ Ophthalmopathy (EUGOGO) recommend intravenous methylprednisolone therapy for first line treatment, some characteristics predicting the response are still inconclusive.

**Aim:**

To study the efficacy of intravenous pulse methylprednisolone (IVMP) in treating dysthyroid optic neuropathy (DON) and to identify factors predicting poor response to the treatment.

**Methods:**

All patients diagnosed with DON between January 2010 and December 2021 at Rajavithi Hospital, Thailand, receiving IVMP 1 g/ day for 3 consecutive days were analyzed. The efficacy at 1 week and 3, 6, 12-months in terms of improvement of best corrected visual acuity (BCVA) and proptosis were compiled.

**Results:**

Of the entire 57 DON cases that received IVMP, 50.9% gained at least 0.2 Logarithm of the Minimum Angle of Resolution (logMAR) at 1 week, and the improvement from initial to 1-week BCVA was 0.63 ± 0.63 logMAR (*p* < 0.001) and the decrease in proptosis was 1.8 ± 1.36 mm (*p* < 0.001). The remaining 23 orbits underwent orbital decompression and were excluded from the long-term efficacy analysis. In the last 12-months’ follow-up time, there was an improvement of BCVA (0.53 ± 0.47 logMAR) and proptosis (0.59 ± 0.66 mm) (both *p* < 0.001). At last visit, there was an improvement of BCVA (0.2 logMAR) and proptosis (2 mm) in 76.5, and 5.9% respectively. Significant predictive factors of poor treatment response were age ≥ 55 years (odds ratio [OR]: 8.28, 95% confidence interval [CI]: 1.368–50.121, *p* = 0.021); longer onset duration before treatment (OR: 5.10, 95%CI: 1.061–24.501, *p* = 0.042); and proptosis at baseline (OR: 9.31, 95%CI: 1.872–46.280, *p* = 0.006). The strongest risk factor for predicting poor response to IVMP was poor initial visual acuity (OR: 10.26, 95%CI: 1.363–77.234, *p* = 0.024).

**Conclusions:**

IVMP is effective for both short- and long-term treatment to improve visual acuity greater than proptosis. Older age, longer disease duration, poor initial visual acuity, and proptotic orbits were identified as risk factors for predicting poor response to IVMP treatment in Thai population. DON patients having those risk factors should be suspected, and treated early with IVMP to preserve their future vision.

**Supplementary Information:**

The online version contains supplementary material available at 10.1186/s12886-023-02789-5.

## Introduction

Dysthyroid optic neuropathy (DON) is a serious manifestation of Grave’s ophthalmopathy (GO), the main extra-thyroid manifestation of Grave’s disease, and affects 4–8% of GO patients with an incidence rate of approximately 0.6–1.3 cases per 100,000 population per year [1–3]. Despite the most serious cause of visual loss in GO, it can be reversible if treated in a timely manner [4]. The most complicated part of the disease is not only diagnostic issues but also treatment concerns [5]. Based on the majority of existing literature, including the expert opinion from the European Group on Graves’ Ophthalmopathy (EUGOGO), the recommended first-line of treatment is high-dose intravenous pulse methylprednisolone (IVMP); if the response is poor, urgent orbital decompression should be performed [6, 7]. Guy et al. [8] reported good efficacy of IVMP in severe DON, and several other studies, mostly conducted in Western countries, have also proved its efficacy [9, 10]. Unfortunately, there are no explainable aspects in terms of characteristics predicting the course of response to IVMP treatment. There are several factors that might affect the response rate proposed by previous researchers: the elderly are prone to being unresponsive to treatment because of their age [5], poor initial visual acuity is identified as a potential factor for poor response [11], tobacco smoking is a well-known negative consequence that has an impact on the immune system or directly affects the individual orbit due to heat [12]. In addition, smoking interfered with the efficacy of immunosuppressants [13, 14]. However, some influencing factors are still controversial in each report including sex, proptosis, associated systemic diseases such as diabetic mellitus [1, 15], and unstable thyroid function [5].

The aims of this study were to evaluate the short- and long-term efficacy of IVMP and to assess factors predicting poor response in DON treatment.

## Methods

We included all DON cases diagnosed at the Ophthalmology department of Rajavithi Hospital between January 2010 and December 2021. Patients in the cohort were aged between 18 and 70 years with no prior treatment with intravenous glucocorticoids and had a diagnosis of DON based on at least two of the following criteria [16]: (i) reduced BCVA of two lines or more; (ii) loss of color vision; (iii) optic disc swelling; and (iv) relative afferent pupillary defect. The evidence of DON based on Giaconi et al. [17] included presence of apical crowding such as enlargement of the extraocular muscles, orbital fat expansion, presence of intracranial fat prolapses, and/or a greater 50% of muscle index on orbital computerized tomography scan (orbital CT) was retrospectively reviewed. The clinical diagnosis of DON was made in the patient without evidence of apical crowding on CT, according to Dayan et al. [16] We excluded cases with active infectious disease, uncontrolled hypertension, uncontrolled diabetes mellitus, liver disease, glaucoma, previous orbital surgery/orbital radiation that can alter the extraocular movements, history of receipt of intravenous glucocorticoids within the previous 6 months, hypersensitivity to IVMP, or follow-up time of < 12 months. The baseline characteristics and laboratory data were retrospectively collected and analyzed.

According to Jeon et al. [18] the IVMP regimen given to DON patients at our center is 1 g of methylprednisolone intravenously for 3 consecutive days. A maintenance dose of glucocorticoid after the course of IVMP consists of an oral prednisolone given for 1 week at 80 mg, which is then tapered by 10 mg per week until a dose of 20 mg is achieved. Then, every week the dose is further reduced by 5 mg until it is discontinued. In our series, the response criterion was defied as an improvement of BCVA after IVMP treatment at 1 week visit (short term efficacy) into three groups as follows; 1) favorable outcome defined as an improvement of BCVA of at least or greater than 0.2 logarithm of angle resolution (logMAR); 2) unfavorable outcome was categorized into less responsive group classified as an improved BCVA of at least 0.1 but less than 0.2 logMAR and unresponsive group defined as there was an improvement of BCVA less than 0.1 logMAR. For long-term efficacy, we assessed the improvement of BCVA at the 3, 6, and 12 months follow up compared to baseline visual acuity and comparative analysis was performed between the groups.

We also assessed the improvement in proptosis in all visits. Patients unresponsive to IVMP were further directed toward orbital decompression surgery and excluded from the long-term efficacy analysis. Thyroid status was determined by laboratory testing for thyroid-stimulating hormone (TSH) and free thyroxine (fT4). TSH levels between 0.270 and 4.200 mIU/L were considered normal, while fT4 levels > 1.7 ng/dl and < 0.93 ng/dl were considered abnormally high and low, respectively.

Based on their laboratory results, all patients with DON were categorized into euthyroid, hypothyroid, or hyperthyroid groups. The relevant factors associated with the poor treatment response were carefully selected from the literature review. Patients were considered to have poor initial visual acuity if their BCVA was ≥1 logMAR. BCVA was measured using a universal Snellen chart and converted to logMAR units for ease of statistical analysis; intraocular pressure (IOP) was measured in mmHg with applanation tonometry. Pressures > 21 mmHg were considered abnormal. Proptosis was measured in millimeters by means of a Hertel Exopthalmometer, and if the value was ≥22 mm, proptosis was positive. Automated visual field was tested with Humphrey Field Analyzer HFA II 750 (Carl Zeiss Meditec Inc., Dublin, CA, USA) using a 30–2 threshold program with the Swedish Interactive Threshold Algorithm (SITA) Fast strategy represented in decibels (dB). Color vision was tested by Ishihara plates and interpreted as abnormal when at least two plates were misread. All visual parameters were measured by ophthalmologists at our center.

Because doses, potency, and duration of glucocorticoids are the main factors contributing to many side effects, this study also collected data on the overall adverse effects during the first-week to the final follow-up visit, including hyperglycemia, gastrointestinal symptoms, Cushingoid features, infection-related to glucocorticoids, elevated liver enzymes, and steroid-induced glaucoma. Recurrent DON was considered if the patient’s visual acuity worsened to ≥0.2 logMAR after initial improvement following IVMP treatment.

### Ethical approval

The study was approved by the Ethics Committee of Rajavithi Hospital (certificate number: 136/2564). All patients provided written informed consent before their data was collected.

### Statistical analysis

All patients who met the inclusion criteria during the study period were recruited into the cohort, and data about the affected eyes were recorded for analysis and expressed either as frequencies or as mean ± SD. Normality of the data was determined with the use of skewness, kurtosis, and Shapiro–Wilk normality test. Thyroid function test was expressed as median ± range or as the 25th and 75th percentiles (interquartile range, IQR). Differences between the baseline characteristics of the treatment groups were compared using the chi-squared and Fisher’s exact test. The Mann–Whitney U test was used for comparison of thyroid function tests, and all continuous data were compared using independent-samples *t*-tests assuming equal variances. The treatment outcomes were compared at the time of first examination and 1 week after receipt of IVMP 1 g for 3 consecutive days in terms of improvement of BCVA and proptosis for short-term efficacy. For long-term efficacy, we compared baseline visual status and 12-month BCVA and proptosis by paired *t*-test. Univariable and multivariable binary logistic regression analysis were employed to study factors predicting poor response of IVMP at the 1-week follow-up period after treatment. Potential predictive factors identified by univariable analysis with *p* < 0.5 were included into the multivariable model. All analysis were performed with SPSS version 25 (IBM Corporation, Armonk, NY, USA).

## Results

A total of 80 orbits from all DON cases (76 patients) were retrospectively reviewed, and four patients had bilateral DON. In case of bilateral DON, the worst side was included in the study. Twenty-three orbits were excluded: seven had a follow-up time of < 12 months, four had been previously treated with orbital radiation (ORT), three had a history of prior intravenous glucocorticoids (IVGC) treatment within the preceding 6 months before diagnosis of DON, and another nine had previously undergone orbital decompression (OD) as initial treatment. A total of 57 orbits (57 patients) fulfilled our inclusion criteria, and their data were used for further analysis. 23 cases (40.35%) underwent orbital CT scan which revealed an enlargement of the extraocular muscles or the presence of fat prolapses via superior ophthalmic fissure (SOF). All 57 DON cases received IVMP 1 g per day for 3 consecutive days and maintenance oral prednisolone as mentioned above. Favorable outcome determined by improvement of BCVA of at least 0.2 logMAR at 1-week follow-up time compared to baseline. As shown in Table 1, there were 29 orbits in the favorable outcome group and 28 orbits in the unfavorable outcome group.

**Table 1:** Baseline clinical characteristics of the orbits of patients with DON based on the treatment outcome^a^

Clinical characteristic	Total	Favorable	Unfavorable	
*n*, (%)	*n* = 57 orbits	(29)	(28)	*p*-value
Age – years (mean ± SD)	50.75 (12.29)	49.10 (11.49)	52.46 (13.05)	0.306
Sex – Female	47 (82.5)	22 (75.9)	25 (89.3)	0.297^†^
Current use of Tobacco	19 (33.3)	11 (37.9)	8 (28.6)	0.454
BCVA – logMAR (mean ± SD)	0.85 (0.63)	0.61 (0.48)	1.09 (0.67)	0.003
Color vision test abnormal				
Abnormal	41 (71.9)	23 (79.3)	18 (64.3)	0.207
Disc morphology				
Normal	32 (56.14)	14 (48.28)	18 (64.29)	
Swelling	18 (31.6)	11 (37.9)	7 (25)	0.294
Pale	7 (12.3)	4 (13.8)	3 (10.7)	1^†^
Proptosis – mm (mean ± SD)	20.55 (3.18)	19.91(3.17)	21.21 (3.1)	0.123
IOP – mmHg (mean ± SD)	18.61 (4.09)	17.55 (3.74)	19.71 (4.21)	***0.045***
Visual field Defects in dB (median [IQR])	−22.6 (−28; −16.4)	−23 (−28; −20)	− 22.25 (−29.25; −10.5)	0.388^‡^
Biochemical and immunological characteristics				
TSH – mU/l (median [IQR])	0.06 (0.006–0.752)	0.098 (0.005–0.845)	0.035 (0.007–0.836)	0.729^‡^
FT4 – pmol/l (median [IQR])	1.51 (1.01–3.16)	1.51 (1.01–3.72)	1.48 (0.95–2.5)	0.549^‡^
Thyroid status				
Euthyroid	24 (42.1)	13 (44.8)	11 (39.3)	0.672
Hyperthyroidism	28 (49.1)	14 (48.3)	14 (50)	
Hypothyroidism	5 (8.8)	2 (6.9)	3 (10.7)	
Type 2 diabetes mellitus	22 (38.6)	8 (27.6)	14 (50)	0.082
Time from onset to treatment in month (median [IQR])	1.3 (0.6–3.7)	1.17 (0.55–3.47)	1.3 (0.61–3.93)	0.457^‡^
Mean follow-up time in month (mean ± SD)	14.81 (4.06)	13.91 (2.63)	15.75 (5.03)	0.088

^a^ The favorable outcome criteria are considered ≥0.2 improved in logMAR. Values are expressed as mean ± standard deviation (SD) or as median and interquartile range (IQR), as appropriate. Percentages may not total 100 because of rounding-off. Time to treatment, and follow up time is represented in months. *IVMP* Intravenous pulse methylprednisolone. *BCVA* Best-corrected visual acuity, *logMAR* Logarithm of the minimum angle of resolution. *IOP* Intraocular pressure. *TSH* Thyroid stimulating hormone. *fT4* Free thyroxine. Bold italicized text indicates statistical significance

† Fisher’s exact test

‡ Mann–Whitney U test

The mean age of all patients was 50.75 ± 12.29 years with no difference between the mean age in the groups (49.10 ± 11.49 years and 52.46 ± 13.05 years in the favorable and unfavorable outcome groups, respectively, *p* = 0.306). Both groups showed female predominance (75.9 and 89.3%, respectively, *p* = 0.297), and there was no difference in the observation of tobacco use between the two groups (*p* = 0.454). Baseline BCVA was 0.85 ± 0.63 logMAR with a statistically significant difference between groups (0.61 ± 0.48 and 1.09 ± 0.67 logMAR in the favorable and unfavorable outcome groups respectively, *p* = 0.003). The majority of DON cases had color vision deficiency, but a difference between the two groups was not significant (*p* = 0.207). Disc morphology, both swelling and pale, was insignificant between the two groups. Proptosis showed an overall mean of 20.55 ± 3.18 mm, which was not significantly different between both groups (*p* = 0.123). There was a marginal difference in baseline intraocular pressure between the two groups (17.55 ± 3.74 and 19.71 ± 4.21 mmHg, respectively, *p* = 0.045). Visual field defect (dB) was not significantly different between the two groups (*p* = 0.388). With regard to an abnormality of thyroid function test, no difference of median (IQR) of either TSH or FT4 in the two groups (*p* = 0.729 and *p* = 0.549, respectively) was noted. Twenty-four patients had euthyroid status (13 and 11 patients in the favorable and unfavorable outcome groups, *p* = 0.672). Almost 50% of patients with DON had hyperthyroidism in our series (48.3% in the favorable outcome group and 50% in the unfavorable outcome group). More patients in the unfavorable outcome group had type 2 diabetes mellitus (DM) than in the favorable outcome group (50% vs. 27.6%, *p* = 0.082). Overall, the median time from onset to treatment was 1.3 months (IQR: 0.6–3.7 months) with a no difference between both groups (*p* = 0.457). In this series, Mean follow up time between favorable and unfavorable outcome was indifferent (*p* = 0.088).

The efficacy of IVMP at the 1-week visit (short-term efficacy) is demonstrated in Table 2. with the improvement rate of BCVA 50.9% (29/57 orbits) while improvement of proptosis was 22.8% (13/57 orbits) in the first week. BCVA in the first week after receiving three consecutive doses of IVMP was 0.63 ± 0.63 logMAR with a difference between the post- and pre-treatment values of 0.21 ± 0.28 logMAR (*p* < 0.001). A significant improvement of proptosis was noted with a difference between post- and pre-treatment values of 1.8 ± 1.36 mm (decrease from 20.55 ± 3.18 mm to 18.75 ± 2.15 mm, *p* < 0.001).

**Table 2:** Visual acuity and proptosis at 1-week after IVMP^a^

Parameters	Total (*n* = 57 orbits)	
(%)		*p*-value
BCVA, logMAR (mean ± SD)		
Baseline	0.85 (0.63)	***< 0.001***
Post-treatment	0.63 (0.63)	
Difference^b^	0.21 (0.28)	
Proptosis (mean ± SD)		
Baseline	20.55 (3.18)	***< 0.001***
Post-treatment	18.75 (2.15)	
Difference^b^	1.8 (1.36)	
Improvement in BCVA – no./total no.^c^	29/57 (50.9)	
Improvement in proptosis – no./total no.^c^	13/57 (22.8)	

^a^Assessment of visual outcomes after 1 week of IVMP treatment. BCVA, best-corrected visual acuity; logMAR, logarithm of the minimum angle of resolution. Bold italicized text indicates statistical significance

^b^At baseline and at 1-week follow-up post-treatment, the mean difference between pre- and post-treatment values were calculated and reported as mean ± standard deviation

^c^Favorable outcome was considered as ≥0.2 improvements in logMAR and reduction in proptosis of ≥2 mm after treatment, for visual acuity and proptosis, respectively

Long-term treatment efficacy of IVMP at 3, 6 and 12-months follow-up time is presented in Table 3. The remaining 34 orbits were analyzed, because 23 orbits were unresponsive (visual acuity did not improve by at least 0.1 logMAR), they were excluded owing to subsequent orbital decompressive surgery. At the 3-month follow-up visit, there was an improvement of BCVA to 0.61 ± 0.62 logMAR (*p* < 0.001) and decrease of proptosis to 18.41 ± 2.17(*p* < 0.001). At the 12-month- follow-up time, a significant difference between post- and pre-treatment values of BCVA and proptosis was noted (0.53 ± 0.47 logMAR and 0.59 ± 0.66 mm, respectively, both *p* < 0.001). The percentage of improved BCVA at 12 months compared with the baseline visit was 76.5% (26/34) and of decreased proptosis was 5.9% (2/34 orbits). No recurrent DON was observed during the 12 -month period in our series.

**Table 3:** Long-term visual acuity and proptosis at 3, 6 and 12 months after IVMP^a^

Parameters	Total (*n* = 34 orbits)	
		*p*-value
BCVA, logMAR (mean ± SD)		
Baseline	1 (0.76)	
Post-treatment (3mo)	0.61 (0.62)	***< 0.001***
Post-treatment (6mo)	0.47 (0.41)	***< 0.001***
Post-treatment (12mo)	0.47 (0.43)	***< 0.001***
Difference^b^	0.53 (0.47)	
Proptosis (mean ± SD)		
Baseline	20.07 (3.19)	
Post-treatment (3mo)	18.41 (2.17)	***< 0.001***
Post-treatment (6mo)	18.38 (2.15)	***< 0.001***
Post-treatment (12mo)	19.49 (2.83)	***< 0.001***
Difference^b^	0.59 (0.66)	
Improvement in BCVA – no./total no.^c^ (%)	26/34 (76.5)	
Improvement in proptosis – no./total no.^c^ (%)	2/34 (5.9)	
Recurrences^d^	0	

^a^Assessment of long-term visual outcomes after 3-,6-, and 12-months of IVMP treatment. Of the 57 patients, 23 were found to be unresponsive to IVMP and had undergone orbital decompression surgery and were thus excluded from the long-term analysis. BCVA, best-corrected visual acuity; logMAR, logarithm of the minimum angle of resolution. The p-value of the comparison between 3-,6-, and 12-months with baseline values is reported. Bold italicized text indicates statistical significance

^b^The mean difference between baseline and 12-months follow-up post-treatment values were calculated and reported as mean ± standard deviation

^c^Favorable outcome was considered as ≥0.2 improvements in logMAR and reduction in proptosis of ≥2 mm after treatment, for visual acuity and proptosis respectively

^d^Recurrences of optic neuropathy was defined as worsening of visual acuity of ≥0.2 in logMAR after a period of normalizing visual acuity or new-onset of worsening color vision, RAPD, and disc swelling

A comparison of baseline characteristics between favorable and unfavorable outcome groups is presented in Table 4. At 1 week, there were 29 patients in the favorable outcome group and 28 patients in the unfavorable outcome groups. The criteria of favorable or unfavorable outcome were based on an improvement of at least 0.2 logMAR as compared to the baseline BCVA. Intergroup comparison of older age (≥55 years) showed no statistical significance (44.8% vs. 67.9%, respectively, *p* = 0.08). A slightly higher proportion of poor initial BCVA in the favorable outcome group (89.7%) was compared with the unfavorable outcome group (67.9%), with no significance (*p* = 0.44); whereas the presence of disc swelling was greater in the favorable outcome group than the unfavorable outcome group (37.9% vs. 25%, respectively; *p* = 0.294). Proptosis was significantly detected in the unfavorable outcome group (*p* = 0.005). The favorable outcome group had a somewhat shorter duration than the unfavorable outcome group from onset to treatment, but this difference was not statistically significant (51.7% vs. 64.3%, respectively; *p* = 0.337). Although there were more euthyroid cases in the favorable outcome group, this difference was not significant (*p* = 0.672). Neither type 2 DM nor smoking was different between the two groups (*p* = 0.082 and *p* = 0.454, respectively).

**Table 4:** Comparison of baseline characteristics between the two groups of treatment outcome^a^

Variable, Total (n = 57 orbits)	Favorable	Unfavorable	
n, (%)	(29)	(28)	*p*-value
Age ≥ 55 years	13 (44.8)	19 (67.9)	0.08
Female sex	22 (75.9)	25 (89.3)	0.297^†^
Smoking	11 (37.9)	8 (28.6)	0.454
Poor baseline visual acuity	26 (89.7)	19 (67.9)	0.44
Disc swelling	11 (37.9)	7 (25)	0.294
Proptosis	11 (37.9)	21 (75)	***0.005***
Euthyroid status	13 (44.8)	11 (39.3)	0.672
Type 2 diabetic mellitus	8 (27.6)	14 (50)	0.082
Duration of vision loss ≥1 month.	15 (51.7)	18 (64.3)	0.337

^a^ The favorable outcome criteria are considered ≥0.2 improved in logMAR after 1 week of IVMP. Prognosis-related factors were selected based on a review of the literature. Percentages may not total 100 because of rounding-off. Cut-off for poor baseline visual acuity is logMAR of ≥1. *IVMP* Intravenous methylprednisolone. *logMAR* Logarithm of the minimum angle of resolution. Bold italicized text indicates statistical significance

† Fisher’s exact test

The comparison of baseline characteristics of the 2 groups based on the improvement of BCVA of < 0.1 logMAR (unresponsive) or ≥ 0.1 logMAR (responsive) at 1 week after IVMP treatment was disclosed (Supplementary Table 1). 31 patients gained at least 0.1 logMAR, and 26 patients were unresponsive (attaining less than < 0.1 logMAR). We found that poor baseline VA, proptosis and type 2 diabetes mellitus were significantly observed in unresponsive group (*p* = 0.027, *p* = 0.018, and *p* = 0.03 respectively). No significant differences in patient age, gender, smoking, disc swelling, euthyroid status, and duration of visual loss were found in the present study.

All 57 patients with DON who received IVMP 1 g for 3 days were included and analyzed in the univariable and multivariable logistic regression model to identify a predictive factor for poor response after IVMP treatment at the 1-week period as shown in Table 5. Older age (≥55 years), female sex, onset duration (≥1 month), disc swelling, poor baseline VA, proptosis (≥22 mm), smoking, and type 2 DM were identified as significant factors in the univariable analysis as follows. Older age had an increased odds ratio (OR) of 2.60 (95% confidence interval [CI]: 0.883–7.645, *p* = 0.083); Female increasing risk for poor treatment outcome with an OR = 2.65 (95% CI: 0.610–11.519, *p* = 0.193); onset duration (≥1 month) increasing risk with an OR = 1.68 (95% CI: 0.581–4.859, *p* = 0.338); and disc swelling had a decreased OR = 0.55 (95% CI: 0.175–1.702, *p* = 0.296). Baseline BCVA≥1 logMAR increased the risk of poor treatment response with an OR = 4.11 (95% CI: 0.978–17.229, *p* = 0.054). Proptosis had a significantly increased risk with an OR = 4.91 (95% CI: 1.574–15.314, *p* = 0.006). Smoking had an OR 0.66 (95% CI: 0.215–1.989, *p* = 0.455). Type 2 DM was identified as a risk factor with an OR = 2.63 (95% CI: 0.873–7.893, *p* = 0.086).

**Table 5:** Univariable analysis of factors predicting unfavorable outcome to IVMP treatment^a^

Variable	Odds Ratio (95% CI)	*p*-value
Age ≥ 55 years	2.6 (0.883–7.645)	0.083
Female sex	2.65 (0.61–11.519)	0.193
Smoking	0.66 (0.215–1.989)	0.455
Poor baseline visual acuity	4.11 (0.978–17.229)	0.054
Disc swelling	0.55 (0.175–1.702)	0.296
Proptosis	4.91 (1.574–15.314)	***0.006***
Euthyroid status	0.92 (0.318–2.643)	0.872
Diabetic mellitus	2.63 (0.873–7.893)	0.086
Onset to treatment time of ≥1 month.	1.68 (0.581–4.859)	0.338

^a^ Unfavorable outcome criteria were considered as < 0.2 improvement in logMAR after 1 week of IVMP. Prognosis-related factors were selected based on a review of the literature. Cut-off for poor baseline visual acuity is logMAR of ≥1. Odds ratios were calculated with the use of logistic regression analysis. Variables with *P* < 0.5 were included in multivariable model. *CI* Confidence interval, *IVMP* Intravenous pulse methylprednisolone, *logMAR* Logarithm of the minimum angle of resolution. Bold italicized text indicates statistical significance

After adjustment of all covariable factors in the multivariable analysis (Table 6), older age (≥55 years) significantly increased the risk of poor treatment response with an OR = 8.28 (95% CI: 1.368–50.121, *p* = 0.021), and long duration of onset (≥1 month) increased the risk approximately 5 times with an OR = 5.10 (95% CI: 1.061–24.501, *p* = 0.042). Proptosis was identified as a potential risk factor with an OR = 9.31 (95% CI: 1.872–46.280, *p* = 0.006). Poor baseline BCVA was the strongest predictive factor of having poor response of IVMP with an OR = 10.26 (95% CI: 1.363–77.234, *p* = 0.024). Female sex, smoking, type 2 diabetes mellitus, and disc swelling were no longer significant in the multivariable analysis. Euthyroid status was not identified as a significant predictive factor of poor treatment response in both the univariable and multivariable analysis in the current study.

**Table 6:** Multivariable analysis of factors predicting unfavorable outcome to IVMP treatment^a^

	Odds Ratio (95% CI)	*p*-value
Age ≥ 55 years	8.28 (1.368–50.121)	***0.021***
Female sex	2.06 (0.330–12.847)	0.439
Smoking	0.68 (0.120–3.884)	0.666
Poor baseline visual acuity	10.26 (1.363–77.234)	***0.024***
Disc swelling	0.37 (0.071–1.97)	0.246
Proptosis	9.31 (1.872–46.28)	***0.006***
Diabetic mellitus	1.08 (0.225–5.153)	0.926
Onset to treatment time of ≥1 month.	5.10 (1.061–24.501)	***0.042***

^a^Unfavorable outcome criteria were considered as < 0.2 improvement in logMAR after 1 week of IVMP. Prognosis-related factors were selected based on a review of the literature. Cut-off for poor baseline visual acuity is logMAR of ≥1. Odds ratios were calculated with the use of multivariable logistic regression model. *CI* Confidence interval, *IVMP* Intravenous pulse methylprednisolone, *logMAR* Logarithm of the minimum angle of resolution. Bold italicized text indicates statistical significance

Cumulative adverse events after IVMP treatment during the 1-week follow-up to the last follow-up visit is shown in Supplementary Table 2. The vast majority of events were hyperglycemia (73.68%) that were spontaneously resolved without medications. Three patients (5.26%) had mild gastrointestinal symptoms (abdominal discomfort and pain). Another three patients (5.26%) suffered from development of cushingoid features; fortunately, there was no concerning long-term cosmetic sequalae. We found that no patients developed steroid-induced glaucoma or serious infection in the series.

## Discussion

Our cohort study showed that the short- and long-term efficacy of IVMP in treating DON was 50.9% in the first week; additionally, we identified that older age (≥55 years), long duration of onset (≥1 month), poor baseline VA (≥1 logMAR), and proptosis at baseline were predictors of poor response to IVMP in the first week.

Intravenous glucocorticoids have been used as the first line of treatment for DON by several previous studies and per the EUGOGO 2021 recommendation [8–10, 19]. The criteria of improvement are varied based on different parameters including BCVA, color vision, or visual field [9, 10, 20, 21]. Our present study set the criteria based on previous studies [8, 9]. We found that IVMP 1 g for 3 consecutive days improved visual parameters with respect to both VA and proptosis. The improvement in vision after 1 week was significantly better than the improvement after 12 months. As corticosteroid efficacy is dependent on the dosage, their actions have processed via genome by binding at glucocorticoid receptors or express via other transcription factors when using lower dose 100 mg and those are long-term effect. For high dose of IVMP acts via non-genome process by acting on decrease number and activity of plasma cell and dendritic cell which is a quick response within 1 day and 8 days [22, 23]. This theory is supported by Bartalena et al. who found recurrence of DON after successful medical treatment in more than 30% patients from 1 week to several months after three consecutive doses of IVMP [24]. Although our study observed no recurrence of DON, the benefit of IVMP slightly decreased in the long-term. In addition, we found no difference in efficacy between the 6 months and at last visit which was similar to Jeon et al. [18], who proposed a stable vison in patients after the 6 months of DON treatment with IVMP and maintenance oral corticosteroid. The improvement in proptosis by at least 2 mm at first week was 22.8% (13/57 eyes) in our study. Our results are consistent with those of another multicenter study which showed that IVGC can improve proptosis by approximately 1 mm in a European population [25].

Aging increases the risk of severity in thyroid eye disease as found by researchers in both Western and Eastern populations [1, 18, 26, 27]. The current study observed that older age (≥55 years) is a predictive risk factor of poor response to IVMP, in line with Miśkiewicz et al’s findings, wherein young age was a positive predictor for visual recovery in Polish patients with DON [28]. Anderson et al. [29] proposed that different ages showed a variety of manifestations in either fat or muscle enlargement, i.e., younger subjects have fat predominance and older subjects have muscle enlargement. In addition, a recent study by Guo et al. [30] discovered that the fat ratio in the orbit is a predictor of good response to IVGC in active GO. Those findings support the idea that young patients tend to have fat predominance and better response to IVMP in our population. However, this result is inconclusive and should be prospectively studied by analyzing both visual and radiological parameters to predict treatment outcome in the future.

Previous studies discovered that duration of disease is a crucial factor influencing the treatment outcome in TED or DON [5, 28, 30]. Miśkiewicz et al. [28] revealed that DON patients presented with 2 months duration gain more response rate compared patients presented with 7 months disease duration. Another study in Asians observed that having symptoms < 1 year obtained more benefits of treatment in active TED (Thyroid eye disease) [30]. Tagami et al. [5] found that a 3-month disease duration showed significantly better visual outcome than a 6.5-month disease duration in DON patients. Similarly, our series observed that longer duration of optic neuropathy significantly increased unresponsiveness to IVMP by approximately 5 times. The explainable theory supported by a study on optic neuritis proposed the benefit of IVMP in the sense that they can relieve inflammation and preserve undamaged nerve fibers [31].

Poor baseline VA was identified as a risk factor for unresponsiveness to IVMP in many studies [5, 28, 30]. Mckeag et al. [32] found mild visual loss had better visual outcome in term of VA after IVGC. Tagami et al. [5] observed that baseline BCVA worse than 0.7 logMAR was more prone to requiring orbital surgery owing to failure of IVMP therapy. Garip Kuebler et al. [11] in their retrospectively review stated that a baseline BCVA of 0.3 logMAR obtained better therapeutic benefit than BCVA of > 0.6 logMAR. Correspondingly, our study observed that poor BCVA ≥1 logMAR increased the odds ratio of unresponsive outcome by 10 times in a multivariable analysis model.

The presence of proptosis in predicting response to IVGC is controversial. Wiersinga et al. [33] proposed that the fibrotic stage in orbital fibroblasts of TED has poor response to corticosteroids. In addition, proptosis may not correlate with DON as proposed by Mckeag et al. [32] In a Chinese population, Guo et al. [30] found that proptosis (> 20.78 mm) was a significant positive factor to the response of IVGC in moderate-to-severe GO. Conversely, the present study found proptotic orbits (≥22 mm) significantly decreased the IVMP response by 9 times. It is difficult to draw a convincing conclusion, considering that Guo et al. [30] studied active GO, while our study was on patients with DON; this may reflect the differences in severity and affect the treatment outcome.

Other predictive factors of poor response were analyzed. Sex, smoking, hyperthyroidism, type 2 DM, and disc swelling were not risk factors in our study. Smoking has a harmful effect on patients with GO due to several reasons. It increases oxygen free radicals in orbital tissues and lowers IL-1 [34, 35]. Eckstein et al. [36] found that smoking is a dose-dependent factor of unresponsiveness to immunosuppressant therapy, but smoking effect is no longer than 1 year. Bartalena et al. [13] proved that tobacco use decreases the efficacy of Fluocortolone; however, it has no impact on the final visual outcome. Recently, Xing et al. [14] discovered that smoking increased the odds ratio by 12.4 to poor response to IVGC in GO; however, they found no difference between current and ex-smokers. Interestingly, smoking was not a significant risk factor in our series. This is likely because the number of smokers in our study was few, which further reduced the analytical power.

Hyperthyroidism and hypothyroidism have been proposed as risk factors for the severity of GO owing to activation of TSH receptors and release of oxygen free radicals [37, 38]. Kung et al. [39] found no relationship between T3 or T4 and severity of GO in an Asian population. Roy et al. [40] and Wang et al. [41] found contradictory results and showed that baseline increase in T4 and euthyroid status affected the efficacy of IVGC in active GO. Recently, a Japanese study discovered instability of thyroid status influenced worse response to IVMP and was more prone to requiring orbital decompression in DON patients [5]. Interestingly, our study found no association of euthyroid status in predicting treatment response. Although Balazs et al. [42] proposed that methimazole has immunosuppressive benefits and patients who received it may gain better therapeutic outcome that those that did not. We did not study the effect or dosage of anti-thyroid medication, because most cases were on thyroid medication prescribed by their primary hospitals, rather than from our center.

Type 2 DM has been significantly observed in GO or DON in several studies [1, 15]. Its impact on final visual outcome is different among each study. Kalmann et al. [43] and Jeon et al. [18] found no difference in visual function. By contrast, a study by Ramamurthy et al. [44] revealed increased prevalence and severity of DM in TED and found that 9 out of 10 patients with DON had type 2 DM. Correspondingly, Rath et al. [45] discovered that DM is a risk factor for DON in the Indian population and tended to have worse final visual outcome. Although type 2 DM was frequently observed in our series, it was not a significant predictor of poor response in the study.

Currò et al. [9] discovered disc swelling at initial diagnosis was a risk factor for unresponsiveness to IVGC in DON patients. Surprisingly, although disc swelling did not reach statistical significance in binary logistic regression analysis, it seemed to be a protective factor of poor response. This explanation may not be the actual biological reason, but it may be because of improved awareness of the ophthalmologist, given that the presence of disc swelling is a very specific finding in DON disease, and it is hence likely that those patients were received earlier diagnosis and treatment than those in the normal disc group [32].

The strength of our study is its large sample size with 12-months follow up time that presents in both short- and long-term efficacy, and reveals the safety of intravenous methylprednisolone with a maintenance dose of oral corticosteroids in treating dysthyroid optic neuropathy. The following are some of the limitations of the study: first, because of its retrospective nature, the outcome of some visual parameters such as color vision and visual field were not studied after the treatment because of missing data. Second, since our referred patients already received anti-thyroid medication from their primary hospitals, we could not study the benefit of methimazole as an immunosuppressive agent. Third, approximately half (34 patients) had no confirmation of apical crowding on orbital CT scan due to economic restriction and received a diagnosis of possible DON based on the clinical finding of Dayan et al. [16] (decreased VA and color vision, presence of RAPD, and disc swelling) This probably affects the diagnostic accuracy of the disease in the present study. However, our study identified older age (≥55 years), longer onset duration before treatment (≥1 month), poor baseline BCVA (≥1 logMAR), and proptosis (≥22 mm) as risk factors for unfavorable treatment response with IVMP in DON in Thai populations.

## Conclusion

Intravenous methylprednisolone is effective for both short- and long-term improvement of visual acuity greater than proptosis. Older age, longer onset duration before treatment, poor initial visual acuity, and proptotic orbit were identified as risk factors for predicting poor response to IVMP in our Thai populations. DON patients having those risk factors should be suspected and treated early with IVMP to preserve their future vision.

## Supplementary Information

**Additional file 1: Supplementary Table 1.** Comparison of baseline characteristics between the two groups of treatment response*. **Supplementary Table 2.** Cumulative adverse events from 1 week to the last follow-up visit.*

**Additional file 2.**

## Data Availability

All data generated or analyzed during this study are included in this published article and its supplementary information files.
