# Supplementary material for: A single-center analysis of visual outcomes and associated factors after intravenous methylprednisolone treatment for dysthyroid optic neuropathy
Source: BMC Ophthalmol. 2023 Jan 23;23:32. doi: 10.1186/s12886-023-02789-5 (PMC9869532; doi:10.1186/s12886-023-02789-5)
Supplement: Supplementary file 1 — Additional file 1: Supplementary Table 1. Comparison of baseline characteristics between the two groups of treatment response*. Supplementary Table 2. Cumulative adverse events from 1 week to the last follow-up visit.* [file 12886_2023_2789_MOESM1_ESM.docx]

Supplementary Table1 Comparison of baseline characteristics between the two groups of treatment response*

| Variable, Total (n=57 orbits) | Responsive | Unresponsive |  |
| --- | --- | --- | --- |
| n, (%) | (31) | (26) | p-value |
| Age ≥55 years | 15 (48.4) | 17 (65.4) | 0.198 |
| Female sex | 24 (77.4) | 2 (88.5) | 0.319^†^ |
| Smoking | 11 (35.5) | 8 (30.8) | 0.707 |
| Poor baseline visual acuity | 3 (9.7) | 9 (34.6) | ***0.027***^†^ |
| Disc swelling | 12 (38.7) | 6 (23.1) | 0.206 |
| Proptosis | 13 (41.9) | 19 (73.1) | ***0.018*** |
| Euthyroid status | 14 (45.2) | 10 (38.5) | 0.610 |
| Type 2 diabetic mellitus | 8 (25.8) | 14 (53.8) | ***0.03*** |
| Duration of vision loss ≥1 month. | 17 (54.8) | 16 (61.5) | 0.610 |

* The treatment response criteria are considered ≥0.1 improved in logMAR after 1 week of IVMP. Percentages may not total 100 because of rounding-off. Cut-off for poor baseline visual acuity is logMAR of ≥1. IVMP, Intravenous methylprednisolone. logMAR, logarithm of the minimum angle of resolution. Bold italicized text indicates statistical significance.

† Fisher’s exact test.

Supplementary Table 2. Cumulative adverse events from 1 week to the last follow-up visit.^*^

| Event (%) | Total (n=57) |
| --- | --- |
| Hyperglycemia | 42 (73.68) |
| Gastrointestinal symptoms^†^ | 3 (5.26) |
| Development of cushingoid features^‡^ | 3 (5.26) |
| Serious infections^§^ | 0 |
| Steroid-induced glaucoma^¶^ | 0 |

* The cumulative adverse events recorded from 1 week to last follow-up visit at 12 months. Hyperglycemia defined as capillary blood glucose >180 milligrams per deciliter (mg/dL).

† This category of minor gastrointestinal related-symptoms included abdominal distention, abdominal discomfort, hiccups, increased appetite, nausea, vomiting.

‡ Defined as the developments of cushingoid features (abnormal body fat redistribution shown as truncal obesity, buffalo hump, and moon face) after receiving intravenous pulsed methylprednisolone.

§ Patients with clinical sepsis and any identifiable organ infection are considered to have a serious systemic infection.

¶ Steroid-induced glaucoma is defined as new-onset elevation in IOP after IVMP.
